# Continuous Talking Face Generation Based on Gaussian Blur and Dynamic Convolution

**DOI:** 10.3390/s25061885

**Published:** 2025-03-18

**Authors:** Ying Tang, Yazhi Liu, Wei Li

**Affiliations:** College of Artificial Intelligence, North China University of Science and Technology, Tangshan 063210, Chinaliuyazhi@ncst.edu.cn (Y.L.)

**Keywords:** talking face generation, two-stage, transformer, renderer

## Abstract

In the field of talking face generation, two-stage audio-based generation methods have attracted significant research interest. However, these methods still face challenges in achieving lip–audio synchronization during face generation, as well as issues with the discontinuity between the generated parts and original face in rendered videos. To overcome these challenges, this paper proposes a two-stage talking face generation method. The first stage is the landmark generation stage. A dynamic convolutional transformer generator is designed to capture complex facial movements. A dual-pipeline parallel processing mechanism is adopted to enhance the temporal feature correlation of input features and the ability to model details at the spatial scale. In the second stage, a dynamic Gaussian renderer (adaptive Gaussian renderer) is designed to realize seamless and natural connection of the upper- and lower-boundary areas through a Gaussian blur masking technique. We conducted quantitative analyses on the LRS2, HDTF, and MEAD neutral expression datasets. Experimental results demonstrate that, compared with existing methods, our approach significantly improves the realism and lip–audio synchronization of talking face videos. In particular, on the LRS2 dataset, the lip–audio synchronization rate was improved by 18.16% and the peak signal-to-noise ratio was improved by 12.11% compared to state-of-the-art works.

## 1. Introduction

**Figure 1:**
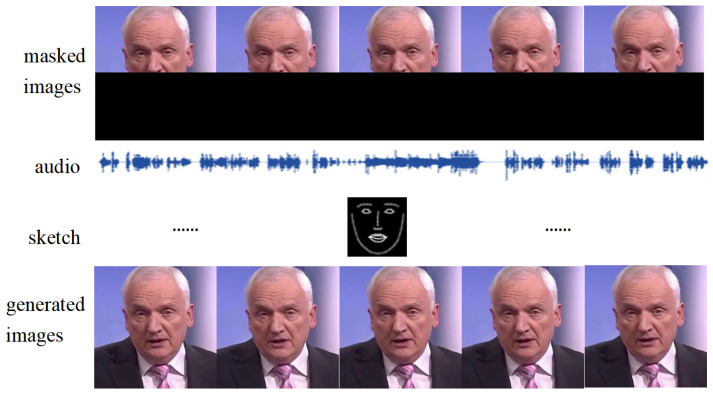
The model first generates sketches of the face based on the audio. The sketches’ mouth shape is synchronized with the audio semantics, then masked images render the face based on the sketches.

With the popularization of cell phones, short videos have become one of the most common forms of entertainment. Short videos are composed of audio and video, and high-quality short videos must achieve audio and video synchronization. For example, when watching the newscast, if the anchor’s mouth shape is not synchronized with her voice, it will greatly affect the viewing experience of the audience. Therefore, audio–video synchronization is crucial for video generation. Talking face generation video is a kind of short video that has been widely used in the field of voice-over and video conferencing. Currently, the hotspot of talking face generation has changed from generating the whole face based on pictures and audio only to generating only the mouth region in the video. In other words, only the lower half of the face needs to be generated, thus ensuring the coherence of head motions in the generated video, as shown in Figure 1.

In the field of talking face generation, there has been significant development in the use of encoder–decoder architecture for end-to-end video generation [1,2,3,4]. Jamaludin et al. [3] used an audio time-series encoder to extract audio features and an image encoder to extract image features, and the two were directly joined together as input features for generating the face. However, this method cannot fully capture the semantic correlations between the features, making some of the mouth shapes inconsistent with the voice. Similarly, the use of intermediate representations for generating talking faces has also seen significant development. Intermediate representations include face keypoints and 3D Morphable Models (3DMMs) [5]. The use of 3DMMs to generate speaking faces [6] is similarly a recently popular approach. In addition, with the continuous advancement of Recurrent Neural Networks (RNNs), Long Short-Term Memory (LSTM), and transformers, the temporal and semantic feature extraction of audio has become better. Some approaches [7,8,9] utilize these models to extract audio features to generate intermediate representations (e.g., face keypoints), then render the intermediate representations to generate faces. In order to improve the quality of face videos further, these features are merged through the attention mechanism. However, these methods are not sensitive to capture subtle facial expression changes, since the correlation between audio and facial expressions is not obvious and requires a complex mapping process. These methods [8,10] generate a face by adding a mask to the lower half of the face area of a reference image and then using a render model to generate the lower half of the face area. However, since the lower half of the face is completely unknown, it is often difficult to keep the generated result corresponding to the original upper half of the face.

Although existing methods have achieved certain success in terms of facial generation quality [11] and the accuracy of lip movements [12,13], they overlook the discontinuity between the generated mouth shapes and the original upper half face in the reference images. Additionally, the generated results often suffer from artifacts, which impact the quality of experience of the generated videos.

Gaussian methods have a wide range of applications [14,15,16,17,18] in a variety of areas. Gaussian blur has been widely applied in image processing [19]; however, it has not been applied to face generation field. By performing weighted averaging on pixel values, Gaussian blur can effectively reduce sharpening artifacts or discontinuities at boundaries in generated images, enabling smooth connection in facial details such as skin texture and edge contours. Furthermore, the multi-scale characteristics of Gaussian blur can be utilized, which can adjust the balance between the local details and global features of the generated images at different resolution levels, making the generated faces more natural and coordinated.

To address the above issues, we propose a two-stage approach comprising a dynamic convolution generator for predicting facial landmarks from audio and a dynamic Gaussian renderer for converting facial landmark sketches into target frames. Our approach aims to generate mouth shapes for the lower part of the face that are synchronized with the audio semantic information. Specifically, in the landmark generation stage, we design a dynamic convolution transformer generator to overcome the limitations of traditional transformer-based landmark generators in capturing fine-grained facial movements. The dynamic convolution transformer generator employs a dual-pipeline parallel processing mechanism, taking audio features, reference images, and posture features as inputs and evenly dividing them into two sub-feature maps along the channel dimension. One sub-feature map is input into the Self-Attention with Local Feature Augmentation (SALA) module, which captures the global long-range dependency information of the driven audio. Another sub-feature map is input into the Dynamic Audio Convolution (DAC) module, which enhances the modeling of local details and spatial dynamics changes. Additionally, the dynamic audio convolution module is capable of dynamically adjusting the convolution kernel weights based on the audio input features to more accurately generate the dynamic landmarks of the lips and jaws. In the video rendering stage, to address the discontinuity between the upper and lower facial areas in the generated images, we design a dynamic Gaussian renderer (adaptive Gaussian renderer) based on Gaussian blur. This module achieves seamless and natural connection by smoothing the boundary between the upper and lower facial areas. To handle the discontinuity between the upper and lower areas of the generated facial images, the dynamic Gaussian renderer employs the Gaussian blur technique. An initial mask is set for the lower facial region, then Gaussian blur is applied to the boundary areas of the mask to achieve natural connection between the upper and lower areas of the generated facial images. The blurred mask is then applied to the reference facial image to generate target facial frames with smooth connection between the upper and lower facial regions. Experiments show that, compared to existing methods, our approach improves the accuracy of mouth movements and the realism of facial videos. Our main contributions are summarized as follows:

- A dynamic convolutional transformer generator is designed using a dual pipeline processing mechanism, which enhances the degree of feature correlation of the input features at the time scale and the ability to model details at the spatial scale, respectively.
- A dynamic Gaussian renderer, designed to ensure continuity between the upper and lower facial regions in the generated images, is based on Gaussian blur and enables seamless and natural connection in the generated facial images.
- We have designed a two-stage method for generating talking face videos. By adopting dynamic convolution and Gaussian blur, our method produces talking face videos with synchronized mouth movements and realism.

We conducted experiments on the LRS2, HDTF, and MEAD neutral expression datasets, demonstrating that our method outperforms existing approaches in terms of realism and lip–audio synchronization.

## 2. Related Work

### 2.1. Audio-Driven Generation of Speaking Faces

Audio-driven talking face generation [20,21,22,23,24,25] is the generation of talking face videos from a given audio clip. The main approaches in recent research can be categorized into two groups: end-to-end direct face image generation and image generation through intermediate representation. Rochow et al. [25] used a transformer to design a model with an encoder and decoder architecture that combines keypoints and facial expression vectors extracted from drive frames for end-to-end generation of character-specific facial dynamics. K R et al. [26] input the lower half of the masked target face as an a priori pose, and the subsequently generated lower half of the face was directly covered onto the original video. Zhong et al. [8] used a similar approach in their translation model by inputting the masked lower half of the target face and using the translation model to generate the lower half of the face, which was directly covered to the original video. These approaches ensure the change in head pose; however, the generated lower half of the face does not match the original video. Intermediate representations can be further categorized into two types, one based on 3DMMs [26,27,28] (3D Morphable Models) and the other based on 2D keypoints [8,24]. The methods proposed by Song et al. [28] and Yi et al. [27] reconstruct 3D facial information directly from 2D facial images, combining 3D Morphable Models (3DMMs) with texture features, angles, and expression parameters to construct 3D facial models. They then generate 2D projected facial images using Generative Adversarial Networks (GANs). However, the methods based on 3D facial modeling rely on 3DMM models and video time-series rescheduling, resulting in the generated face videos being unnatural. The study by Ji et al. [29] integrates emotionally relevant facial dynamics into acquired motion representations by learning key-point actions from audio and through linearly additive displacements. This approach effectively integrates audio-driven keypoint dynamics and emotional displacements to generate expressive face effects. The model mainly focuses on the dynamic generation of facial expressions without head pose variations, which can lead to a lack of visual realism in the rendered faces.

### 2.2. Landmark-Based Talking Face Generation

In research on landmark-based talking face generation, methods that map speech features to landmarks [7,8] have been widely utilized. Face keypoints are detected [30] using detection tools. Nian et al. [31] used a parallel multi-branch network architecture to map input speech features to facial landmark sequences. While this approach combines multi-branch networks and landmark sequence generation techniques, it lacks precise modeling for complex facial movements such as non-linear lip dynamics and jaw motions. Suwajanakorn et al. [7] utilized Recurrent Neural Networks (RNNs) to learn the mapping from audio input to lip features and then synthesized high-quality lip textures. They relied on Long Short-Term Memory (LSTM) models [32] to learn the mapping from audio to landmark sequences. Zhong et al. [8] designed a transformer-based landmark generator to generate lip and jaw landmarks from audio. This generator effectively leveraged prior landmark features of the speaker’s face, ensuring that the generated landmarks were accurately aligned with the facial contours of the speaker. While transformer architectures are effective at modeling long-term temporal dependencies, their handling of rapidly changing details and short-term dynamics in speech audio is less comprehensive, which can lead to reduced precision in complex speech-driven scenarios. Many works [33,34] have developed various methods to enhance the performance of transformers, which are effective in improving accuracy.

## 3. Methods

### 3.1. Face Keypoint Generation Stage

**Figure 2:**
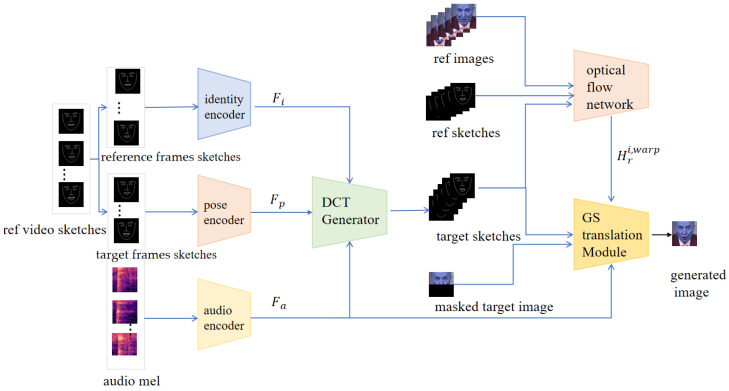
Overall structure of the model.

The two-stage model proposed in this paper includes a face keypoint generation stage and a face keypoint rendering stage, as shown in Figure 2. The model in the facial keypoint generation stage is the Dynamic Convolution Transformer (DCT) generator, which consists of a transformer that incorporates a dynamic convolutional network to effectively capture the complex features of the audio input. The model in the facial keypoint rendering stage is the adaptive Gaussian renderer. In the adaptive Gaussian rendering stage, the model introduces Gaussian blur technology to achieve adaptive fusion of image and audio features. To enhance the realism and quality of the generated images, the model combines visual quality discrimination loss and L1 reconstruction loss.

#### 3.1.1. Preprocessing Audio and Video

Given the audio *A* and the video frames *V* of the reference image, our goal is to generate a video of a speaking face with the same mouth shape as the audio semantics using the face of the reference image. In the audio processing section, the audio $A=\{a_{1},\ldots ,a_{n}\}$ is processed into a 16×80 Mel spectrogram, respectively, i.e., h=16, w=80, and the spectrogram is fed to an audio encoder to extract audio features $F_{a}\in R^{d}$. The 16 Mel bands cover the main frequency range of the human voice, while 80 time steps provide sufficient time resolution to capture the dynamics of the audio signal. In the video processing section, the video is extracted frame by frame. Then, *T* target frames $T_{F}=\left\{t_{1},t_{2},\ldots ,t_{T}\right\},t_{i}\in R^{128\times 128\times c}$ are randomly selected from the video. *N* reference frames $I=\left\{r_{1},r_{2},\ldots ,r_{N}\right\},r_{i}\in R^{128\times 128\times c}$ are randomly selected from the remaining video frames, which exclude the target frames. The reference images are then concatenated and input into an identity encoder to extract identity features $F_{s}$. At the same time, the target frame is input into the pose encoder to extract pose features $F_{p}$.

(1) $$F_{s}=f_{s}\left(I\right),\;F_{s}\in R^{d}$$

(2) $$F_{p}=f_{p}\left(T\right),\;F_{p}\in R^{d}$$

Here, $f_{s}\left(\cdot \right)$ and $f_{p}\left(\cdot \right)$ denote the identity encoder and pose encoder, respectively, and *d* is the dimensions of both features.

#### 3.1.2. Dynamic Convolutional Transformer Generator

**Figure 3:**
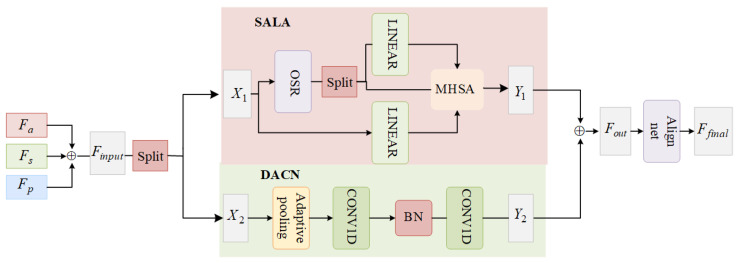
DCTGenerator structure.

To improve the adaptability of facial expression poses, we designed a dynamic convolutional transformer generator (DCTGenerator). Compared to directly using a transformer-based generator, our DCTGenerator combines the Self-Attention and Localized Feature Augmentation (SALA) module and a Dynamic Audio Convolutional Network (DACN). SALA utilizes a multi-head attention mechanism that captures information from different subspaces in parallel when processing pose features, audio features, and reference face keypoints, resulting in a more comprehensive understanding of the complex relationship between audio features and face keypoints. DACN focuses on local optimization processing for in-depth analysis of features for face region, which is especially important for accurately generating key face motions such as mouth movements and lower forehead movements. This is shown in Figure 3.

Audio features, identity features, and posture features are concatenated along the temporal dimension to obtain $F_{\mathrm{input}},F_{\mathrm{input}}\in R^{B\times T\times d}$. Input features $F_{\mathrm{input}}$ are split into two parts, $X_{1}$ and $X_{2}$, along the channel dimension. This process includes using larger overlapping blocks to better represent spatial structures near block boundaries. The SRA specifically helps in refining feature representation by focusing on important spatial details within the overlapping blocks, enhancing the accuracy and integrity of feature representation. After processing $X_{1}$ using the SALA module, we obtain the feature map $Y_{1}$. The sub-feature $X_{2}$ is fed into the DACN module, which includes a convolutional layer that dynamically adjusts its parameters based on the input features. DACN produces output feature map $Y_{2}$. Then, $Y_{2}$ and another feature map $Y_{1}$ are concatenated along the channel dimension to form the combined feature $F_{\mathrm{out}}$. The combined feature $F_{\mathrm{out}}$ is then input into a dimension alignment network, resulting in the final facial keypoint feature representation $F_{\mathrm{final}}$.

(3) $$F_{\mathrm{input}}=\left\{X_{1}\in R^{B\times T\times \frac{d}{2}},X_{2}\in R^{B\times T\times \frac{d}{2}}\right\}$$

(4) $$Y_{1}=E_{\mathrm{SALA}}\left(X_{1}\right),\;Y_{1}\in R^{B\times T\times \frac{d}{2}}$$

(5) $$Y_{2}=E_{\mathrm{DACN}}\left(X_{2}\right),\;Y_{2}\in R^{B\times T\times \frac{d}{2}}$$

(6) $$F_{\mathrm{out}}=Y_{1}⊕Y_{2},\;F_{\mathrm{out}}\in R^{B\times T\times d}$$

(7) $$F_{\mathrm{final}}=E_{\mathrm{align}}\left(F_{\mathrm{out}}\right),\;F_{\mathrm{final}}\in R^{B\times T\times d}$$

We use dynamic convolution to combine multimodal information from audio, pose landmarks, and reference landmarks to generate dynamic convolutional weights that correspond to the input features.

Finally, the face keypoint $F_{\mathrm{final}}$ feature representation is segmented into two parts based on different regions, which are used to generate keypoints for lips and jaws, respectively. These segmented embeddings are converted into facial keypoints through two mapping functions. These two parts are then concatenated to obtain the facial keypoints (m) of the final predicted audio semantic content. These facial keypoints are drawn to the target sketch according to the facial linking rule.

#### 3.1.3. Loss Function for Face Keypoint Generation

Accurately locating face keypoints during training is critical to the overall facial analysis. By promoting sparsity and increasing robustness to outliers, L1 loss can help models locate these critical points more accurately rather than simply averaging all prediction errors. During the training process, we use the L1 reconstruction loss to train the predicted landmark keypoints:

(8) $$L_{1}=\frac{1}{T}\sum_{t=1}^{T}\left(||{\hat{m}}_{t}^{i}-m_{t}^{i}{||}_{1}+{||{\hat{m}}_{t}^{j}-m_{t}^{j}||}_{1}\right)$$

where ${\hat{m}}_{t}^{i}$ and ${\hat{m}}_{t}^{j}$ denote the ground-truth landmark keypoints of the lips and jaws, respectively.

In order to enhance the smoothness of the time series, we introduce a continuity regularization method that constrains the predicted landmark to ensure smoother variations. The equation is as follows:

(9) $$L_{c}=\frac{1}{T-1}\sum_{t=1}^{T-1}\left(||\left({\hat{m}}_{t+1}^{i}-{\hat{m}}_{t}^{i}\right)-\left(m_{t+1}^{i}-m_{t}^{i}\right){||}_{2}+{||\left({\hat{m}}_{t+1}^{j}-{\hat{m}}_{t}^{j}\right)-\left(m_{t+1}^{j}-m_{t}^{j}\right)||}_{2}\right)$$

The overall training loss for generating landmarks based on audio is as follows:

(10) $$L=L_{1}+\lambda_{c}L_{c}$$

where $\lambda_{c}$ is the constant 1.

### 3.2. Face Keypoint Rendering Stage

#### 3.2.1. Landmark and Image Alignment Phase

First, the predicted lip and jaw keypoints are concatenated with the original keypoints of the face with pose features to sketch the target face $S_{t}$. Then, in order to generate a continuous face image, the sketch of the reference image at the t-th frame, the sketches of the prior k frames, and the sketches of the subsequent k frames are input as the basis for predicting the face image at the t-th frame. In addition, reference images $I_{r}$ and the sketch $S_{r}$ provide the face pose features. The sketches $S_{r}$ were made by extracting the face keypoints from this reference image.

#### 3.2.2. Dynamic Gaussian Renderer

**Figure 4:**
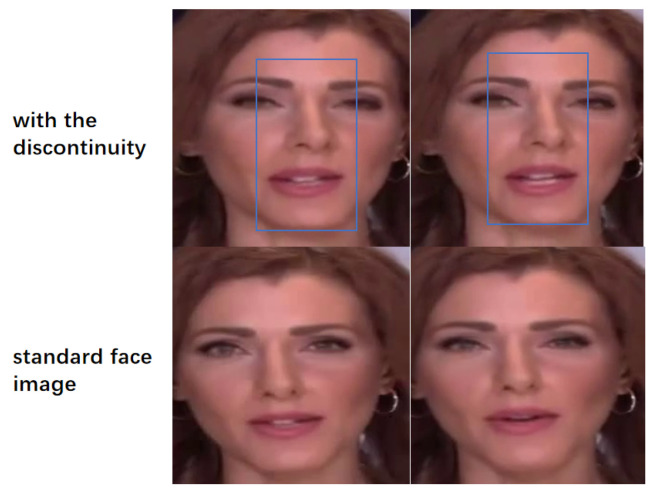
The generated lower half of the face image exhibits visual discontinuity with the upper half of the original input face image. The discontinuity between the mouth shape of the generated face and the original video is marked in the blue box, seeing that the generated mouth shape does not align with the original nose.

In order to solve the problem of visual discontinuity between the generated lower half of the face and the upper half of the face of the original input face, see Figure 4. This paper introduces a dynamic Gaussian renderer based on a Gaussian blur guiding mechanism, aimed at optimizing the generation of the lower half of facial images. Specifically, a mask of the same size as the target facial image is first initialized by setting the value of its lower half region to 0, as described by the following formula:

(11) $$M\left(x,y\right)=\left\{\begin{array}{cc} 0, & \mathrm{if}\;y<\frac{h}{2}\\ 1, & \mathrm{if}\;y\geq \frac{h}{2} \end{array}\right.$$

In this context, M(x,y) represents the mask values, and *h* is the height of the image. The mask is then blurred using a Gaussian blur operation to generate a smooth transition blur mask.

(12) $$M_{\mathrm{blur}}=G*M$$

Here, *G* is the Gaussian kernel and ∗ denotes the convolution operation. The kernel size of the Gaussian blur is set to 5 × 5, and the value is fixed to 1.0. These parameters are determined through a series of pre-experiments to optimize the balance between visual effects and performance. The Gaussian blur is mainly applied to the mask region, i.e., the lower part of the face, to smooth the transition region and to reduce hard boundaries in the composite image.

The blur mask gradually reduces the influence of the input image. Finally, the blur-guided image $I_{\mathrm{mask}}$ is combined with the target sketch $S_{r}$ to generate a new target sketch, $S_{t}^{\mathrm{new}}\in R^{3\times H\times W}$, which is used as the input for the GS translation module.

We input the multi-frame reference images ${\left\{I_{i}\in R^{3\times H\times W}\right\}}_{i=1}^{N}$, their corresponding sketches ${\left\{S_{i}\in R^{3\times H\times W}\right\}}_{i=1}^{N}$, and the k frames, together with the prior k frames and subsequent k frames of the target face sketch $S_{t-k:t+k}$, into the optical flow network to obtain the optical flow motion field ${\left\{\mu^{i}\in R^{2\times H\times W}\right\}}_{i=1}^{N}$. We added an output layer after the optical flow network to predict the weights $\omega_{i}\in R^{H\times W}$ of the reference image $I_{r}^{i}$. The aggregated warping features $H_{r}^{i,\mathrm{warp}}$ were then calculated based on the weights and the aggregated warping features (h) at spatial resolution. The formula is as follows:

(13) $$\mu^{i}=G_{\mathrm{optical}\;\mathrm{flow}}\left(I_{r}^{i},S_{r}^{i},S_{t-k:t+k}\right),\;i=1,2,\ldots ,N$$

(14) $$I_{r}^{i,\mathrm{warp}}=\frac{\sum_{i=1}^{N}\omega_{i}\mu^{i}\left(I_{r}^{i}\right)}{\sum_{i=1}^{N}\omega_{i}}$$

where $\mu^{i}\left(I_{r}^{i}\right)\in R^{3\times H\times W}$ is the image warped by the motion field. Specifically, the optical flow computation is achieved by combining a convolutional network and a SPADE (Spatially Adaptive Normalization) block. Reference images ${\left\{I_{i}\in R^{3\times H\times W}\right\}}_{i=1}^{N}$ and corresponding reference sketches ${\left\{S_{i}\in R^{3\times H\times W}\right\}}_{i=1}^{N}$ are combined to form the $Fl_{input}$. This step is performed by concatenating the images and sketches in the channel dimension, generating the input feature dimension as (B∗T,6,H,W). $Fl_{input}$ begins with feature extraction using two convolutional layers, conv1 and conv2, which gradually decrease the spatial resolution of the feature map while increasing the depth, from 128 × 128 to 64 × 64. The SPADE block is then used to adjust the convolved feature map to fit the dynamic input (the driving sketches $S_{t-k:t+k}$). The SPADE block uses the driving sketches $S_{t-k:t+k}$ as an additional conditional input, which helps to ensure that the output features are spatially consistent with the driving sketch. The optical flow ${\left\{\mu^{i}\in R^{2\times H\times W}\right\}}_{i=1}^{N}$ and the corresponding weights $\omega_{i}\in R^{H\times W}$ are output through the final convolutional layers conv_4 and conv_5, respectively. These outputs describe how each pixel point of the image should move and the importance of those movements. The reference image is warped to the target position using the optical flow output. This is achieved through the warping function, which adjusts the image content according to the predicted optical flow.

(15) $$H_{r}^{i,\mathrm{warp}}=\frac{\sum_{i=1}^{N}\omega_{i}\mu^{i}\left(h\right)}{\sum_{i=1}^{N}\omega_{i}}$$

Here, $\mu^{i}\left(h\right)\in R^{3\times H\times W}$ is the visual feature warped by the motion field.

The input to the Gaussian translation module consists of a target sketch $S_{t}^{\mathrm{new}}$, reference features $H_{r}^{\mathrm{warp}}$, and a Gaussian blurred facial image $I_{\mathrm{mask}}$. The module generates the final face image $I_{t}$ by combining the audio features $A_{t}$ through conv, SPADE, and AdaIN operations.

(16) $$I_{t}=G_{r}\left(S_{t}^{\mathrm{new}},H_{r}^{\mathrm{warp}},I_{\mathrm{mask}},A_{t}\right)$$

#### 3.2.3. Loss Functions for Rendering Modules

The perceptual loss of this model calculates the $L_{1}$ distance between the activation maps of the pre-trained VGG-19 network. The warp loss $L_{w}$ between the aggregated warped image $I_{r}^{i,\mathrm{warp}}$ and the actual image $I_{r}^{i}$ is used to constrain the alignment module to calculate an accurate motion field, as described by the following formula:

(17) $$L_{w}=\sum_{i}{||\phi_{i}\left(I_{r}^{i,\mathrm{warp}}\right)-\phi_{i}\left(I_{r}^{i}\right)||}_{1}$$

where $\phi_{i}$ represents the activation map of the ith layer of the VGG19 network. Reconstruction loss $L_{r}$ and style loss $L_{s}$, which are similar in structure to $L_{w}$, are utilized to compute the statistical errors between the VGG-19 feature maps, with the goal of reducing the discrepancies between the generated face $I_{t}$ and the real face $I_{r}$.

(18) $$L_{r}=\sum_{i}{||\phi_{i}\left(I_{t}\right)-\phi_{i}\left(I_{r}\right)||}_{1}$$

(19) $$L_{s}=\sum_{i}{||G_{i}^{\phi }\left(I_{t}\right)-G_{i}^{\phi }\left(I_{r}\right)||}_{1}$$

Here, the Gram matrix $G_{i}^{\phi }$ is constructed from the activation maps $\phi_{i}$. The model utilizes reconstruction loss, warp loss, and style loss. The formula is as follows:

(20) $$L=\lambda_{w}L_{w}+\lambda_{r}L_{r}+\lambda_{s}L_{s}$$

In the experiments, we set $\lambda_{w}=2.5$, $\lambda_{r}=4$, $\lambda_{s}=1000$.

### 3.3. Experiments

#### 3.3.1. Experimental Setup

In the experiment, we used 2 × NVIDIA A100 GPUs and PyTorch 2.3.1 to build the network models. The specific training parameters are shown in Table 1. The batch size was set to 32 for the audio to keypoint stage, and the model was trained using the Adam optimizer with the learning rate set to $1\times 10^{-4}$, training for a total of 1500 epochs. The batch size was set to 16 for the keypoint rendering to the face image stage, and the model was trained using the Adam optimizer with the learning rate set to $1\times 10^{-4}$, training for a total of 20 epochs. In the data preprocessing stage, we used consecutive five-frame images as the target images. In order to reduce the model’s dependence on a specific reference image during the generation process, we adopted a randomization strategy, i.e., we randomly selected five frames from the reference image as the input. This strategy allowed the model to be trained in a variety of contextual environments, which helped to enhance its generalization ability and thus effectively improved the robustness of the model. The sampling rate used for audio processing during training was 16,000 Hz and the processed audio was mono. The sound resolution of the audio was 16 bits, which effectively ensured audio quality while avoiding unnecessary data bloating. The resolution of the image was 128 × 128, and RGB color resolution was used to ensure the accuracy and naturalness of the rendering.

#### 3.3.2. Datasets

We evaluated the model quantitatively and qualitatively on the LRS2, HDTF, and MEAD datasets. The LRS2 dataset consists of 48,164 video clips from BBC TV outdoor programs. The length of the speech content of each video does not exceed 100 characters. The dataset is divided into training, validation, and test sets with a ratio of 8:2:1. The HDTF dataset consists of about 430 field videos with a resolution of 720 P or 1080 P. In our experiments, we randomly selected 20 videos for testing. The MEAD dataset, on the other hand, contains about 40 h of lab-recorded emotion videos, all at a 1080 P resolution. Considering that our study did not involve visual dubbing of emotional expressions, we specifically selected 1920 videos showing neutral emotions and front perspectives to constitute the MEAD-Neutral dataset.

#### 3.3.3. Evaluation Metrics

We use the Peak Signal-to-Noise Ratio (PSNR) and Structural Similarity Index (SSIM) to evaluate the clarity and realism of the generated video frames. In addition, we used LSE-D and LSE-C to evaluate the synchronization between audio and video. LSE-D represents the distance score between audio and video, which is used to measure the average error between lip shape and audio features. The lower the score, the higher the audio and video match. LSE-C represents the confidence score. The higher the score, the higher the synchronization rate between lip shape and audio. These two metrics were calculated by loading the pre-trained SyncNet model. Finally, we introduced Learned Perceptual Image Patch Similarity (LPIPS) to measure the perceptual difference between the generated video frame and the source video frame.

### 3.4. Results

#### 3.4.1. Quantitative Results

We first conducted experiments on the test set of LRS2 and compared them with four advanced methods. As can be seen from Table 2, our method performed best in both the SSIM and PSNR image quality metrics, and it also achieved the best results in the lip synchronization metrics. In the PSNR metric, our method improved the result by 12.11% compared to the sub-optimal method Wav2Lip, and in the LSE-C metric, our method improved the result by 18.16% compared to the sub-optimal method PC-AVS. This shows that our dynamic convolution transformer generator and dynamic Gaussian renderer fix the discontinuity between the upper and lower half of the face while maintaining visual quality, significantly improving the lip synchronization rate. The metrics of the videos generated using our method are close to the metrics of the real videos, indicating that the differences between the generation results and the real videos are small. The dynamic convolutional transformer generator finely generates mouth shapes based on audio semantics by making specific adjustments to the lip region of each image frame. At the same time, the dynamic Gaussian renderer introduces a Gaussian mask during the rendering process, which not only enhances the overall harmony of the image but also optimizes the visual effect of the transition areas. The effectiveness of this approach is demonstrated through comparison with real video metrics. As can be seen from the metrics in Table 2, the quality of the generated video is extremely close to that of the real video, which indicates that our method is able to effectively enhance the realism of the synthesized video without sacrificing naturalness.

To verify the generalization ability of the model, we compared the final model with three advanced methods on the high-resolution HDTF and MEAD-Neutral datasets. The input data consisted of audio and pose sequences extracted from each test video. As can be seen from Table 3, our model achieved the best results on the lip sync metrics LSE-C and LSE-D, and it also performed well based on the SSIM and PSNR metrics. It is worth noting that our method was not trained on the HDTF dataset, but the test results on the HDTF data were excellent, which shows that our model has strong generalization ability. Similarly, for the untrained dataset, EDTalk and FSRT give poorer results on both datasets. Our method improves the PSNR metric by 30.06% over EDTalk due to the dynamic convolutional transformer generator that better incorporates the features of the audio and the Gaussian blur dynamic renderer, generating the face more naturally when rendering the face and improving the generalizability.

#### 3.4.2. Qualitative Comparison

**Figure 5:**
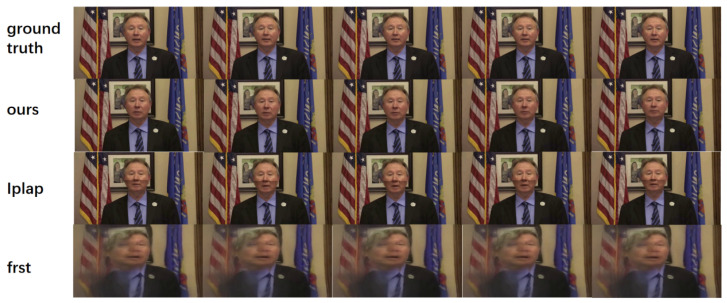
Comparison with several other methods on the HDTF dataset.

We conducted a qualitative comparison with two advanced methods on the HDTF dataset, and the results are shown in Figure 5. Our method demonstrated the best performance in terms of image quality and lip synchronization. As can be seen from Figure 5, the IPLAP method produced a noticeable discontinuity when the lower half of the generated face was concatenated with the original upper half, resulting in an unnatural connection appearance. Meanwhile, the FSRT method showed poor image quality on unknown datasets, indicating a lack of generalization capability.

### 3.5. Ablation Experiments

**Figure 6:**
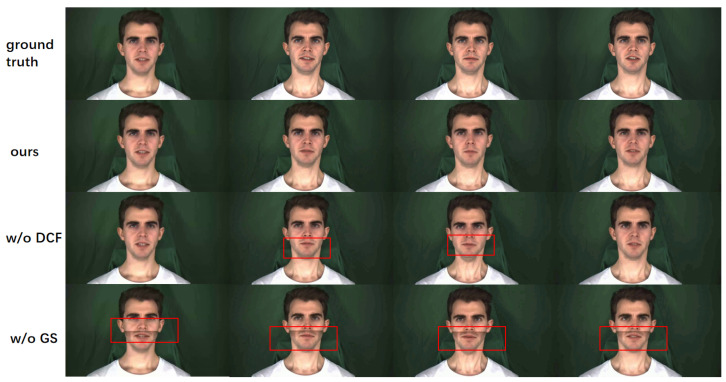
Comparison of ablation experiments. The red box in w/o DCF indicates that the mouth shape is out of sync with the ground truth. A clear line of demarcation can be seen in the red box of w/o GS.

To validate the effectiveness of each module in our method, we conducted the following two ablation studies. First, we removed the dynamic convolution transformer module from the dynamic convolution generator and instead used a standard transformer to integrate audio and facial landmarks. As shown in Table 4, there was a significant decrease in the lip synchronization metric, indicating that the dynamic convolution module plays a crucial role in generating facial landmarks that are well-synchronized with the audio. Furthermore, as seen in Figure 6, removing the dynamic convolution module resulted in a noticeable decrease in the semantic synchronization between the generated lip movements and the spoken audio. This observation suggests that the introduction of dynamic convolution by the transformer enhances the overall learning ability of the model, especially when highly dynamic and complex data need to be processed.

Next, we replaced the Gaussian blur with a simple mask. According to the results in Table 4, it can be seen that the quality metrics of the generated images were significantly degraded. Meanwhile, it can be observed from Figure 6 that a clear demarcation line appeared between the lower half of the face and the upper half of the face. This observation shows that the Gaussian blur effectively reduces hard boundaries between image regions through its superior edge smoothing ability, thus providing more natural visual transitions. This enhances the naturalness and coherence of the image, which is extremely important in facial image processing.

## 4. Conclusions

In this paper, we propose a two-stage talking face generation method that significantly improves the ability to capture complex facial motions by integrating a dynamic convolution module and a dual-pipeline parallel feature processing mechanism to the traditional transformer-based landmark generator. Additionally, to address the discontinuity between the upper and lower parts of the generated face, we designed a dynamic Gaussian renderer based on Gaussian blur. By smoothing the transition at the upper and lower boundaries, seamless and natural transitions are achieved, thereby enhancing the realism of the generated images. Experimental results show that our method produces talking face videos with superior realism and lip synchronization compared to existing methods.

## Figures and Tables

**Table 1:** Training parameters.

Stage	Batch Size	Learning Rate	Optimizer	Epochs
Audio to keypoint	32	$1\times 10^{-4}$	Adam	1500
Keypoint to face image	16	$1\times 10^{-4}$	Adam	20

**Table 2:** Comparison with other methods on the training data LRS2. ↑ indicates that the higher one is better, ↓ indicates that the lower one is better.

Method	SSIM ↑	PSNR ↑	LSE-D ↓	LSE-C ↑
ATVG [11]	0.3944	11.55	8.223	5.584
Wav2Lip [4]	0.8962	27.92	7.789	6.386
PC-AVS [30]	0.4867	15.75	7.101	6.928
FSRT [25]	0.8644	25.50	9.120	4.092
Ours	0.9332	31.30	5.309	8.186

**Table 3:** Comparison of methods on HDTF and MEAD-Neutral datasets. The top dataset is HDTF, and the bottom is MEAD-Neutral. ↑ indicates that the higher one is better, ↓ indicates that the lower one is better.

Method	SSIM ↑	PSNR ↑	LPIPS ↓	LSE-D ↓	LSE-C ↑
ATVG [11]	0.7315	20.5430	0.1104	8.4222	7.0611
Wav2Lip-192 [4]	0.8487	27.6561	0.1208	8.0912	6.9509
PC-AVS [30]	0.6383	20.6301	0.1077	11.4913	3.0293
EDTalk [35]	0.7505	22.0442	0.3674	10.9544	2.4573
FSRT [25]	0.6774	17.7222	0.5051	11.0702	1.9514
Ours	0.8692	30.8394	0.0906	6.3196	8.3954
ATVG [11]	0.8325	23.9723	0.0869	8.8908	5.8337
Wav2Lip-192 [4]	0.8036	25.6863	0.1302	7.8426	6.8515
PC-AVS [30]	0.7754	23.6950	0.0960	6.5035	8.6240
IP-LAP [8]	0.8109	29.2186	0.0979	7.7351	6.2937
EDTalk [35]	0.8469	22.8718	0.1641	7.6053	5.6232
FSRT [25]	0.8892	23.3672	0.1665	14.132	0.1143
Ours	0.9109	29.7470	0.0889	5.2856	8.9076

**Table 4:** Ablation experiment data for each module. ↑ indicates that the higher one is better, ↓ indicates that the lower one is better.

Method	SSIM ↑	PSNR ↑	LSE-D ↓	LSE-C ↑
w/o DCF	0.8783	28.16	7.882	4.796
w/o GS	0.8740	27.28	6.648	8.568
Ours	0.9272	30.08	5.309	8.186

## Data Availability

The original data provided for this study are available on LRS 2 (https://www.robots.ox.ac.uk/~vgg/data/lip_reading/lrs2.html (LRS2 website) accessed on 12 October 2024), HDTF (https://gitcode.com/gh_mirrors/hd/HDTF/tree/main/HDTF_dataset (HDTF on GitCode) accessed on 18 October 2024), and MEAD (https://drive.google.com/drive/folders/1GwXP-KpWOxOenOxITTsURJZQ_1pkd4-j (MEAD on Google Drive) accessed on 18 October 2024) in the public domain.
